# Camera-LiDAR Fusion Method with Feature Switch Layer for Object Detection Networks

**DOI:** 10.3390/s22197163

**Published:** 2022-09-21

**Authors:** Taek-Lim Kim, Tae-Hyoung Park

**Affiliations:** 1 Department of Control and Robot Engineering, Chungbuk National University, Cheongju 28644, Korea; 2 Department of Intelligent Systems & Robotics, Chungbuk National University, Cheongju 28644, Korea

**Keywords:** deep learning, sensor fusion, object detection

## Abstract

Object detection is an important factor in the autonomous driving industry. Object detection for autonomous vehicles requires robust results, because various situations and environments must be considered. A sensor fusion method is used to implement robust object detection. A sensor fusion method using a network should effectively meld two features, otherwise, there is concern that the performance is substantially degraded. To effectively use sensors in autonomous vehicles, data analysis is required. We investigated papers in which the camera and LiDAR data change for effective fusion. We propose a feature switch layer for a sensor fusion network for object detection in cameras and LiDAR. Object detection performance was improved by designing a feature switch layer that can consider its environment during network feature fusion. The feature switch layer extracts and fuses features while considering the environment in which the sensor data changes less than during the learning network. We conducted an evaluation experiment using the Dense Dataset and confirmed that the proposed method improves the object detection performance.

## 1. Introduction

Autonomous vehicles perform convenience functions by judging whether the vehicle recognizes the surrounding environment in situations that may occur while driving. In many cases, two or more sensors are fused in order to implement convenient functions. These convenient functions are called ADAS and are implemented using the camera, LiDAR, and radar. ADAS include LKAS (lane-keeping assist system), BSCW (blind-spot collision warning), and SCC (smart cruise control). To implement these, object recognition and distance estimation functions are essential. In general, the object detection of ADAS is implemented using range-measured LiDAR, radar, and a camera with a lot of data for recognition [1]. Object recognition and distance estimation are possible through sensor fusion [2]. This sensor fusion can realize robust detection results [3]. Sensor fusion is used for various ADAS functions as well as object detection. Object detection is being studied on a network basis, and it is necessary to study a sensor fusion method for network fusion.

Sensor fusion in an autonomous vehicle aims to realize robust functioning by supplementing the weakness of a single sensor with another sensor using multiple data. Sensor fusion can be divided into cases where different modals and the same physical quantity must be connected. The same physical quantity can solve the malfunction in one sensor, reducing uncertainty and realizing robust performance. The fusion of different modals leads to different types of results using different data types [4]. Research on cameras and LiDAR is being actively conducted for use as a sensor for object detection, and it is necessary to think about how to fuse different data [5,6,7,8].

Network-based sensor fusion can be divided into early, intermediate, and late fusion. The advantages of each sensor fusion method are as follows. According to the existing deep-learning network structure, early fusion of the sensor fusion method is easy to implement. Intermediate fusion improves performance by structurally connecting the networks of each sensor [8] and sharing sensor features or separating roles [5,6,7]. The late fusion method uses data from one sensor to suggest a region, and another sensor classifies it [9]. Sensor fusion based on deep learning rather than object detection focuses on different expressions from a modal rather than a structural point of view. We considered a reasonable method for sensor fusion among early, intermediate, and late fusion. For this purpose, it has been found that sensor fusion in different fields mainly uses intermediate methods and focuses on different expressions in the same situation [10,11]. Therefore, we chose an intermediate structure that can fuse different modals.

Previous research has focused on effectively fusing data representations from different sensors when the network learns [12,13]. We investigated the situation in which the camera and LiDAR data change for effective fusion. This paper analyzed the effect of humidity on the point cloud using two types of LiDAR [14]. Heinzer [15] proposed a method to solve this problem by filtering the point cloud based on CNN as a follow-up study. This paper can predict the change in LiDAR data under high humidity conditions. Likewise, the camera will have difficulty recognizing objects in high humidity conditions. For example, the camera tries to learn in a foggy situation by converting it into a clear image through dehazing [16,17,18]. However, it is not easy to apply because the expression methods of the two sensors are different. Methods of fusing various expressions have mainly been studied in fields such as VQA (visual question answering). This paper presented a method to fuse multiple modals effectively [19]. This paper proved that the performance of two or more datasets could be effectively improved with a module with a small amount of computation. Inspired by that study, we designed the network layer for effective data fusion.

The above studies [14,15,16,17,18] confirmed that each sensor responds differently in specific situations. Therefore, if the fusion is performed without considering the sensor’s specific situations, the noise will be reflected and adversely affect network learning. We thought that to solve this problem, the camera–LiDAR sensor fusion method of should differentiate the situation and influence network learning differently. Our research focuses on configuring the module so that when learning by fusion of different data from camera–LiDAR, the sensor’s influence can be given differently considering the surrounding environment. That is, creating a network structure with different dominance.

In this paper, the Feature Switch Layer (FSL) switch module selects important features by applying channel attention to the camera and LiDAR sensor convolution features [20]. When applying channel attention, the channel size was adjusted considering the increase in noise of each sensor. Next, a multimodal transfer module (MMTM) was added to fuse the features for each channel based on the work of Joze et al. [19]. After applying MMTM, spatial attention was applied. Finally, concatenation with the existing feature map generates a recalibrated feature map. The *Both* module is added considering the case where two sensors are robust. Through this, a layer that can learn by reflecting the sensor’s noise was constructed. Each module is written as *Camera* or *LiDAR* dominance module, and if both sensors are robust or weak, use *Both* dominance modules. The network learns features by dominance module. After testing the FSL, a comparative experiment was conducted to contrast the module configuration and improve the performance of the learning method by extracting features for each situation.

The contributions of this paper are as follows.

1. This paper proposes a learning method by selecting a feature to be learned by considering the sensor character. Object detection performance was improved by selecting different features to learn from cameras and LiDAR networks according to these conditions. The experimental results confirmed that the camera supplements the LiDAR sensor in the daytime and foggy conditions, while the LiDAR sensor supplements the camera’s object detection at night.
2. The paper proposes an FSL that selects major features by applying the channel attention of features extracted from the backbones of different networks in the object detection network. Through this layer, the object detection performance is improved without being biased by the situation. Moreover, a method for fusing both features rather than using only one was suggested by combining different ratios.

The rest of the paper is organized as follows. Section 2 explores related studies about object detection, sensor fusion methods, and detection in adverse weather conditions. Section 3 describes the FSL, the overall network structure, and the learning strategy. Section 4 outlines the experiments conducted using the Dense Dataset [21] to evaluate the FSL, including comparing the developed module to the attention module and MMTM [19]. Section 5 concludes the study.

## 2. Related Work

This paper analyzed the effect of dust on LiDAR data [22]. This paper implemented dust and rain conditions in an indoor environment. The distance accuracy and intensity of the objects measured using LiDAR were analyzed by adverse weather conditions. Another paper analyzed the noise of LiDAR data measured in dust clouds [23]. Autonomous driving requires examining how data changes when measuring vehicles and people in adverse weather. Recently, a published study further analyzed the changes to data measured in fog and rain conditions (that is, when the humidity in the air is high) in terms of subjects such as people and cars [14]. Heinzer et. al.’s studies show that LiDAR’s intensity for cars and people or LiDAR’s point-cloud density decreases in fog and rain situations. In adverse weather, the point-cloud-based recognition study was solved by changing the network structure or input expression by analyzing data characteristics [15,24,25,26].

In the past, object detection has been performed by changing the strategy during the day and in nighttime to solve problems in object detection. Typically, vehicle detection at night is different from daytime detection, as it detects taillights or headlights and performs vehicle detection using robust feature extraction [27]. Image enhancement has been studied in the image processing field, and the detection performance has been improved [28]. As an image enhancement technology, the GAN-based dehaze method was studied to remove the noise of the camera image in fog conditions [16,17,18]. Previous studies show that the camera is greatly affected by changes in light, and the direction of problem-solving changes depending on conditions such as day, night, and fog.

Attention is described as seeing an essential element in an image rather than the whole, as with human perception [29]. Recently, an attention mechanism has been applied to network learning and attempted to improve the performance of CNNs, and there are techniques for viewing essential features [30]. Seeing an essential feature of learning means that the network becomes robust to noise and performance. An essential feature in the network is the core of the attention mechanism, and it is also used as a tool to understand why learning is successful [31].

The fusion of camera–LiDAR for object detection focuses on decoupling the sensor’s role [5,6,7]. The multi-stage process used in existing camera–LiDAR fusion it is divided into a method of proposing a 2D candidate region [4,5,32] and a method of proposing a 3D candidate region [6,8]. Network-based sensor fusion includes the VQA and audio-visual speech enhancement (AVSE) fields. The network for sensor fusion in VQA and AVSE is multi-modal. This paper considers how to fuse the extracted features by inputting the network. When different data types such as camera, text, and sound are extracted using a network, it is necessary to learn by adjusting the features when the network is learning [33,34]. The network for sensor fusion must control the reflection of each piece of data.

A squeeze and excitation network (SENet) [35] interprets the network as a channel relationship. SENet inspired MMTM, and MMTM fuses different features. SENet also influenced CBAM [20], which proposed channel attention. Channel attention and spatial attention were proposed by Woo, S et al. [20] and make learning more efficient by allowing us to see important areas according to tasks. Our study designed a module using MMTM and CBAM. Using this, the features are fused in the intermediate. Through this, we tried to achieve fusion between robust networks and features.

## 3. Feature Switch Layer

In this paper, we propose a feature switch layer to teach the dominance of the camera–LiDAR sensor differently. The feature switch layer includes a switch module designed to learn by selecting a feature during learning. First, the network architecture is explained.

### 3.1. Network Architecture

The network architecture consists of a camera and LiDAR backbones. The LiDAR network backbone generally uses a grid method that implements a point cloud based on PointNet [36] or VoxelNet [37]. In this paper, LiDAR data are expressed and fused in the same coordinate system as the camera. When LiDAR point cloud, which is unstructured data, is fused using voxels or raw point cloud, early fusion cannot be performed because the coordinate system is different. In intermediate fusion, it is not easy to match the feature coordinates of two sensors in object detection. Therefore, three-channel data were created by projecting the point cloud to the camera coordinate system. Depth, height, and intensity were used to compose each channel’s data and a three-channel image [38].

**Figure 1:**
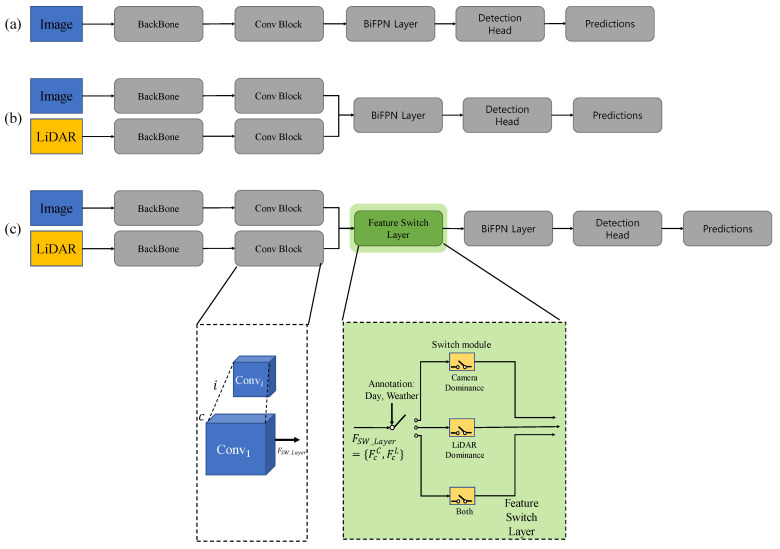
EfficientDet-based network structure. (**a**) EfficientDet [39]; (**b**) EfficientDet fusion method intermediate level; (**c**) EfficientDet with feature switch layer (proposed).

Figure 1 shows the proposed network and the previous network structure [39]. Each sensor’s data is input into the backbone network, which uses EfficientNet [40]. The convolution feature output from each layer is expressed in (1).

(1) $$F_{camera}=\left\{F_{1}^{c},F_{2}^{c},\cdots ,F_{i}^{c}|camera\;backbone\;feature\right\},\;F_{LiDAR}=\left\{F_{1}^{L},F_{2}^{L},\cdots ,F_{i}^{L}|LiDAR\;backbone\;feature\right\}$$

Here, i is 5. By using five-layer features, the features were fused to the switch module and used as the input of the BiFPN layer.

For each sensor feature, *F* is the camera and LiDAR. F is $F\in {\mathbb{R}}^{W\times H\times Channel}$, where *W* and *H* are spatial dimensions, and the Channel is the number of channels extracted from the backbone. *F* changes the size of the spatial dimensions and channels through a convolution operation. The output from the convolution block is represented in (2). Since the output in the backbone layer is different, declare convolution blocks as applicable.

(2) $$F_{c}^{C}=\left\{F_{c1}^{C},\cdots F_{ci}^{C}\right\},\;F_{c}^{L}=\left\{F_{c1}^{L},\cdots F_{ci}^{L}\right\}\;$$

*C* stands for camera, and *L* stands for LiDAR. c is the channel, and c1 is the first operation of the c channel convolution block.

### 3.2. Feature Switch Layer

The feature switch layer (FSL) teaches the network by selecting advantageous sensor features for learning by filtering the data from the sensor that may contain noise. The feature map extracted from the previous backbone network and annotation information for day/night and weather is input as a layer input. The annotation information about day and night, weather, and the dominance of the feature switch module is selected, and the final BiFPN layer input is determined. *Day* is included as day and night, and *Weather* is included as snow, fog, clear, and dense fog.

The input of FSL is as Equation (3). FSL is divided by dominance, and this paper proposes three types: *Camera, LiDAR*, and *Both*. The switch module declares the number of feature maps i for each layer.

(3) $$F_{SW\_Layer}=\left\{F_{c}^{C},F_{c}^{L}\right\}\;\;$$

The switch module is designed to produce different outputs using the inputs in Equation (3). The module design aimed to reflect less noise from each sensor and fuse different features. This will be explained later in the Experimental Section.

**Figure 2:**
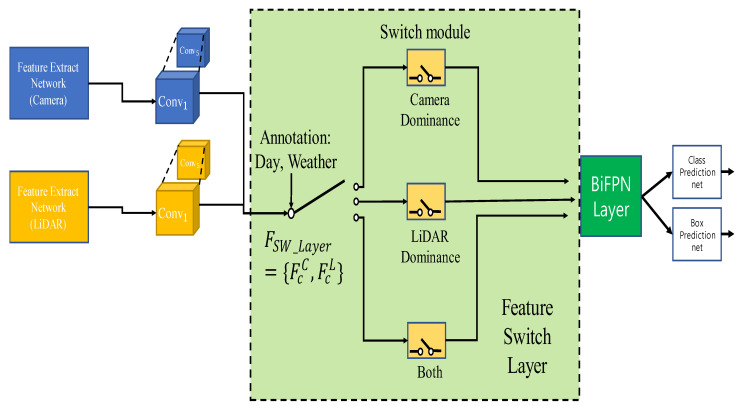
Structure of the feature switch layer when five convolution block features are declared. In the feature switch layer, each sensor and both sensors are declared to have different influences for each sensor.

Determine the input of the feature switch layer through a rule. The rules are detailed in the Section 4.2 Experimental Setups. Figure 2 shows the overall structure of the proposed method. First, a convolution operation is performed on the features extracted from the backbone. The layer has a switch module for each sensor. Through this structure, it is possible to create a structure that takes the influence of the sensor differently by extracting and reinforcing the features of a specific sensor through an attention mechanism. For example, the feature switch layer, in which the camera has dominance, is designed to pay more attention to camera features.

### 3.3. Switch Module

**Figure 3:**
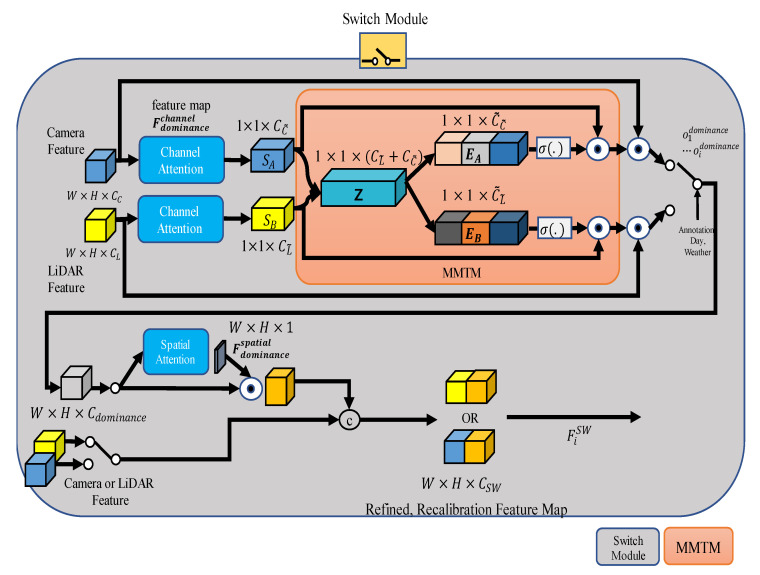
Structure of network with a feature switch module. CBAM [20] and MMTM [19] are applied to each input, and the recalibrated features are selected and processed using annotation.

Simply using concatenation is unsuitable as a sensor fusion method when considering the noise according to the situation, as the network can learn noise as well. Figure 3 shows the structure of the switch module. First, the switch module gives channel attention to the input feature map. Next, through MMTM, features are selected by dominance, and finally, after spatial attention, concatenation with input occurs. A detailed explanation is given one paragraph at a time below.

Channel attention proceeds with squeeze and excitation after pooling. We determined the type of pooling method and squeeze and excitation rates differently for each dominance module. This paper used max pooling and average pooling Equations (4) and (5) for the channel attention module.

As a result of channel attention to camera features, $C_{\tilde{C}}$ applies avg-pooling and max-pooling in the case of the *camera* dominance module. $C_{\tilde{L}}$, the channel attention result of LiDAR features, applies only max-pooling to create feature maps with different channel sizes. In the LiDAR dominance module, the opposite is applied, so that the channel size $C_{\tilde{C}}$ is smaller than $C_{\tilde{L}}$. By changing the pooling method, features of different sizes can be created and fused. In addition, using max-pooling, only robust features can be seen when the sensor is weak.

(4) $$C_{\tilde{C}}=\{\;Camera\;Dominance,\;Both\;:\;AvgPool\left(F_{ci}^{C}\;\right)+MaxPool\left(F_{ci}^{C}\right)\;LiDAR\;Dominance\;:MaxPool\left(F_{ci}^{C}\right)\;\},\;for\;i$$

(5) $$C_{\tilde{L}}=\{\;LiDAR\;Dominance\;:\;AvgPool\left(F_{ci}^{L}\;\right)+MaxPool\left(F_{ci}^{L}\right)\;Camera\;Dominance,\;Both\;:MaxPool\left(F_{ci}^{L}\right)\;\},\;for\;i$$

After pooling for each channel, channel attention proceeds with squeeze and excitation [38]. The paper’s squeeze ratio is called r, and it is taken differently for each *camera* and *LiDAR* dominance module. For this, the ratio of squeeze and excitation must be determined, which is also determined according to Equations (6)–(8) for each dominance module. We vary this ratio according to dominance.

(6) $$F_{\;dominance}^{channel}=\{\;Camera\;Domaince\;:\;r_{camera}>r_{LiDAR},\;for\;i\;$$

(7) $$LiDAR\;Dominance\;:\;r_{camera}<r_{LiDAR},\;for\;i\;$$

(8) $$Both\;:\;r_{camera}=r_{LiDAR},\;\;for\;i\}$$

As a result of channel attention to $F_{dominance}^{channel}\in {\mathbb{R}}^{W\times H\times Channel}$, 1 × 1 × $C_{\tilde{C}}$ or $C_{\tilde{L}}$ is multiplied by each sensor’s feature again to restore the data form as $F_{dominance}^{channel}\in {\mathbb{R}}^{W\times H\times Channel}$. However, we used raw data $1\times 1\times C_{\tilde{C}}\;or\;C_{\tilde{L}}$.

The results of channel attention were merged into a concatenation of the channel attention results. The process of MMTM is shown in Equations (9) and (10). Here, [·, ·] represents the concatenation operation.

(9) $$Z\;=\;W\left[S_{A},\;S_{B}\right]+\;b,$$

(10) $$E_{A}=W_{A}Z+b_{A}\;,E_{B}=W_{B}Z+b_{B},$$

$1\times 1\times C_{\tilde{C}}$ is calculated with the activation function and the existing feature, and it is calibrated and output. As a result, $1\times 1\times {\tilde{C}}_{\tilde{C}}$ and $1\times 1\times {\tilde{C}}_{\tilde{L}}$ are output as $1\times 1\times C_{\tilde{C}}$ and $1\times 1\times C_{\tilde{L}}$ by the MMTM formula, and the result comes out through the activation function. Features $E_{A}$ and $E_{B}$ recalibrated by the MMTM are selected for each case. ⊙ is the channel-wise multiplication. The result is expressed as $o^{dominance}$. In the *both* dominance module, the number of convolution channels of the camera and LiDAR was the same, so j was separated. In this paper, j was set to 3. The output of $o^{dominance}$ using annotation is as shown in Equations (11)–(13).

(11) $$o_{i}^{dominance}=\{Camera\;Domaince\;:\left\{W\times H\times {\tilde{C}}_{\tilde{c}}\right\},\;for\;i\;$$

(12) $$LiDAR\;Dominance\;:\left\{W\times H\times {\tilde{C}}_{\tilde{L}}\right\},\;for\;i$$

(13) $$Both\;:\;\left\{W\times H\times {\tilde{C}}_{\tilde{c}}\right\},\;for\;0<j<i\;,\;\left\{W\times H\times {\tilde{C}}_{\tilde{L}}\right\},\;f\;\left\{else\right\}for\;i\}$$

The output spatial attention of the switch module is called $F_{dominance}^{spatial}$. $F_{dominance}^{spatial}$ has two declared camera and LiDAR domains. Concatenation was carried out by selecting the opposite of dominance. The result is expressed by Equation (14). For example, for the *camera* dominance module, the calibrated camera feature becomes a concatenation $W\times H\times {\tilde{C}}_{C}$ and $W\times H\times C_{L}$. $C_{SW}$ is the result of combining ${\tilde{C}}_{C}$ and ${\tilde{C}}_{L}$. In *LiDAR* dominance module, calibrated LiDAR feature becomes a concatenation $W\times H\times {\tilde{C}}_{L}$ and $W\times H\times C_{C}$. $C_{SW}$ is the result of combining ${\tilde{C}}_{L}$ and $C_{C}$. For a *Both* module, camera dominance is selected by j, and *LiDAR* dominance module is selected for the rest.

(14) $$F_{i}^{SW}=W\times H\times C_{SW}=\left\{\left[F_{dominance}^{spatial},o^{not\_dominance}\right]\right\},\;for\;i$$

$F_{i}^{SW}$ is the final output. The output is used as the input of BiFPN, after which the network configuration is the same as EfficientDet.

## 4. Experiment

The Dense Dataset [14] was used in these evaluations, and an experiment for each fusion method was first conducted to prove the network effect and then evaluated. The metric of the experiment was evaluated using the MS-COCO metrics [41].

### 4.1. Network Model

**Figure 4:**
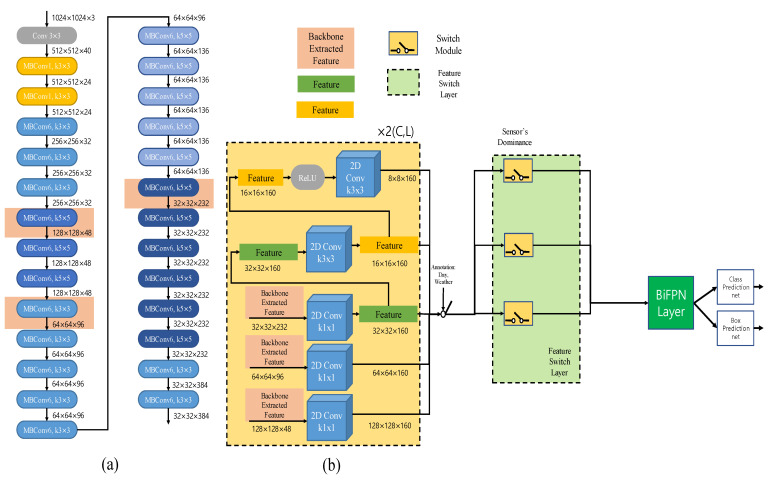
(**a**) Illustration of the EffecientNet-B3 architecture. We used backbone with EfficientNet-B3. (**b**) Input of switch module. C stands for camera backbone operation and L stands for LiDAR backbone operation, and each conv block of the same size is used.

Our deep-learning model is shown in Figure 4a. A general Efficiencynet-b3 was used and the input of the switch module is as shown in the figure. The backbones of the camera and LiDAR are used, respectively. We performed 2D object detection using input to the network. Figure 4b: the input of the switch module. Perform 2D convolution on features extracted as backbones. Since i was set to 5 in the experiment, we used 5 2d convolutions as shown in Figure 4b. Backbone features come out for each camera and LiDAR, and FSL input is determined by annotation. The details of the determination of the input are described in the next section.

### 4.2. Experimental Setups

We used the Dense Dataset. In the Dense Dataset, there are 12,000 samples of stereo camera, Velodyne 64ch LiDAR, radar, and infrared camera in bad weather and situation data. This dataset includes fog, snow, and rain as adverse weather, and includes day and night, so it can be used as a research dataset for adverse weather. In this dataset, data with changes in humidity and light are collected. We used it to verify that discriminative learning can improve object detection performance. The Dense Dataset conducts the training, testing, and validation classifications in clear weather, while the rest of the weather data are not separate datasets. Therefore, the training, validation, and testing datasets were separated in a 7:1:2 ratio, and the experiment was conducted and evaluated. Table 1 shows the number of data sets.

A computer with an Intel Core i7 processor and NVIDIA GeForce RTX 3090 graphics card was used, and the PyTorch library was utilized for training, validation, and testing. The model’s learning rate was set to 0.001, and the minibatch size was fixed to 2 for the previous and proposed methods. Both methods ran until the neural network repeated 50 epochs of the entire training dataset.

The object detection network was trained to detect people and vehicles and exclude buses, trucks, and bicycles. Detection performance was then evaluated based on the camera image, which is the area where both sensors were detected, to proceed with the evaluation of sensor fusion.

**Figure 5:**
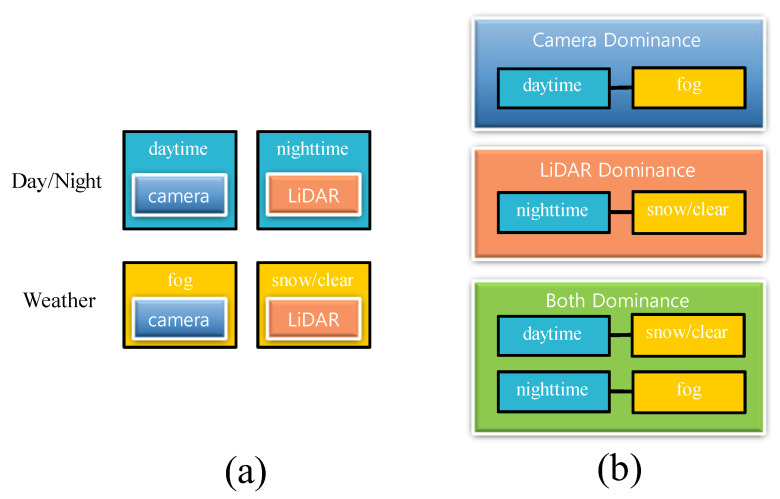
Day and night, weather-dependent sensor dominance. (**a**) Each sensor represents a robust situation. (**b**) Dominance is used in experiments and establishing rules based on the robust sensor.

EfficientDet is the result of using only the camera image. We determined the dominance of camera data based on day and night. Previous studies have shown that LiDAR errors occur in foggy situations [20]. The *LiDAR* dominance module determines based on fog, and the *Both* dominance module considers cases where both sensors are usual or weak. Both sensors are weak during nighttime foggy conditions, and both sensors usually work during the day and in clear or snowy conditions. Figure 5 shows the rules that set the layer. Among the various situations, only the daytime fog situation remained. Fog affects the camera and LiDAR, but considering the daytime point, the daytime and the foggy situation were categorized into *camera* dominance module. In the further explanation using Figure 5, if it is daytime and snowy, both sensors are dominant, so the *Both* module is selected.

We conducted an experiment by increasing the squeeze ratio of the sensor features that we consider important. The experiment parameters are $r_{camera}$ = 16 and $r_{LiDAR}$ = 8 for the *camera* dominance module. Each parameter is a rate of the squeeze. The *LiDAR* dominance module was determined as $r_{camera}$ = 8, $r_{LiDAR}$ = 16, and Both modules were set as $r_{camera}$ = 8, $r_{LiDAR}$ = 8. MMTM also has a squeezing process, with 12 for the *Camera* dominance module and 16 for the *LiDAR*; *Both* dominance modules are used for the squeeze ratio in this paper.

### 4.3. Result

Table 2 shows based on the highest mAP in experiments. The proposed method gives good results in most situations. The performance of object detection in the test dataset is improved by 0.013 compared to the existing method. It is the same or improved by 0.015 in clear and snowy conditions at night. We found that the proposed method is effective by analyzing the results covered by the *Both* dominance module. Achieving performance improvement in both modules is challenging without effectively merging two different modals. The proposed method effectively fuses sensors to achieve performance improvement compared to the existing method in the situation. However, the existing method was still good in day and fog situations. Object detection results should be the same even after repeated training multiple times. In order to check whether the performance of the proposed method is always low, we averaged the results of the top 5 and prepared Table 3.

Table 3 expresses the top 5 results as mAP, average recall, and F1 score. Table 3 shows the results of evaluating the test set by storing the model weights based on the time when the evaluation of learning is best in the validation set. Looking at Table 3, the average value of the proposed method in the top-5 experimental results is high in all metrics. Also, when the variance values are compared, the variance of the proposed method is the smallest, so learning can be performed stably. If the variance value is small, it can be predicted that the training results of the network will be equally good. Our goal was to design a layer for robust object detection. The experimental results show that the proposed method made the network fusion of camera–LiDAR more effective.

Table 4 shows the computational amount of the proposed method as the complexity of the network. When a module is added, the amount of computation is higher than that of a network using only a single sensor. Through the model complexity evaluation, it can be confirmed that the performance of the switch module can be improved by a slight increase in parameters and calculation amount compared to the existing method. The proposed method effectively improved the performance without a significant increase in complexity.

Table 5 determines how many switch modules in the feature switch layers are reasonable. There are only two modules: the dominance module of each sensor. Here, the rule is the determining factor in dominance. As shown in Figure 5a, we created a rule to determine the dominance using the time of day (day or night). Among the rules of the two modules, day and night are designed to learn camera dominance during the day and LiDAR dominance at night. The two modules, fog/not fog, make the camera learn LiDAR if it is not foggy. Table 5 shows the experimental results according to the division of dominance. The intermediate method performs better if the dominance is divided incorrectly in the proposed method. Through the experimental results, we confirmed that learning by dividing the dominance into three types is a robust and improved method for object detection performance.

Table 6 shows the object detection performance according to the module configuration. Channel refers to the result when applying only channel attention in a convolutional block attention module (CBAM). Only channel attention was applied, and three dominance modules were used for the learning strategy. Learning with three dominances by applying only channel attention performed better than adding other modules.

If the module is not used efficiently, it can only be seen that the complexity increases, and the object detection performance deteriorates. The experimental results show that using the channel attention results is crucial. The importance of channel attention was confirmed, and the value described in Section 4.2 produced the best result because of several experiments by changing the hyperparameter.

**Figure 6:**
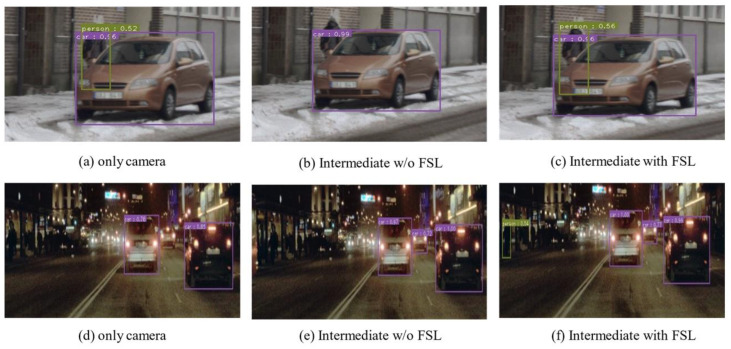
Clear/daytime, nighttime detection results. (**a**–**c**) Clear/daytime, only camera, intermediate w/o FSL, intermediate with FSL (proposed). (**d**–**f**) Clear/nighttime, only camera, intermediate w/o FSL, intermediate with FSL (proposed).

Figure 6 shows each detection result according to Table 2. Figure 6a,d shows the results of learning using only camera images. Figure 6b shows that if fusion is not effective, the performance can be degraded. Figure 6c effectively fuses with the proposed method so that no object learned from the camera is missed. At night, both sensor fusion methods performed better than the camera.

**Figure 7:**
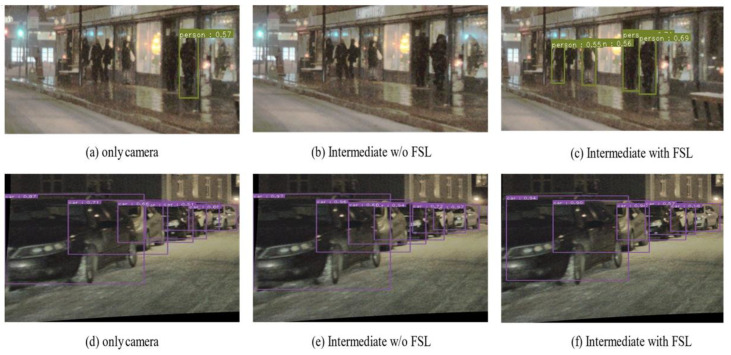
Snow, fog/nighttime detection result. (**a**–**c**): Snow/nighttime, only camera, intermediate w/o FSL, intermediate with FSL (proposed). (**d**–**f**): Fog/nighttime, only camera, intermediate w/o FSL, intermediate with FSL (proposed).

Figure 7 shows the results of snow and fog conditions at night. The intermediate method could not be detected in snowy conditions in some cases. In the case of fog, the detection without FSL was higher than that of the camera, but the bounding box was inaccurate. The proposed method showed robust object detection performance through the experimental results regardless of various situations.

## 5. Conclusions and Future Work

This paper proposes a feature switch layer and an effective sensor fusion method in adverse weather conditions during the daytime and nighttime. The experiments showed that the feature switch layer’s sensor fusion method is more robust than the simple concatenation method. In addition, the effects of different learning methods on performance improvement in deep learning were discovered by analyzing the sensor characteristics according to the weather and day/nighttime. However, in the proposed method, learning is only possible by annotating the weather, and switching by grasping and learning the dominance by itself was not implemented.

Future studies will be directed toward improvements so that the network can detect the weather and vary the network weight by changing the switch module. In addition, we plan to experiment by applying the above network module to 3D object detection. We are collecting various weather and day and night data to confirm the performance improvement in the low channel, which we will use in our next study.

## Figures and Tables

**Table 1:** Number of datasets.

	Step	Training	Validation	Testing
Weather				
Camera Dominance	Daytime, Fog	525	69	140
LiDAR Dominance	Nighttime, Clear	1343	409	877
	Nighttime, Snow	1720	240	480
Both Dominance	Daytime, Clear	2183	399	1005
	Daytime, Snow	1615	226	452
	Nighttime, Fog	525	69	140
Total		8238	1531	3189

**Table 2:** Performance according to weather and performance comparison with other networks. The best performance index among the results of five experiments. D: daytime, N: nighttime, C: clear, F: fog, S: snow.

Network	Camera	LiDAR		Both			Total mAP@[.5]
	D, F	N, C	N, S	D, C	D, S	N, F	
EfficientDet	0.398	0.331	0.375	0.382	0.400	0.342	0.367
EfficientDet w/o FSL	0.469	0.402	0.436	0.409	0.444	0.407	0.414
EfficientDet with FSL	0.448	0.417	0.436	0.422	0.448	0.434	0.427

**Table 3:** Mean and variance of the results of five experiments as average precision, average recall, and F1 Score.

Network	Top5-mAP	Top5-Recall	F1-Score
EfficientDet	0.347 ± 0.00073	0.253 ± 0.00083	0.293 ± 0.00015
EfficientDet w/o FSL	0.398 ± 0.00018	0.309 ± 0.00013	0.348 ± 0.00082
EfficientDet with FSL	0.406 ± 0.00016	0.317 ± 0.00006	0.356 ± 0.00009

**Table 4:** Comparisons of model size and complexity. FLOPs: floating-point operations., PN: parameter number.

Architecture	Sensor Fusion Method	Input Data	FLOPs	PN
EffcientDet	None	1024 × 1024 × 3 (camera)	46.8 G	13.7 M
	Intermediate	1024 × 1024 × 6 (Camera, LiDAR)	180.8 G	33.3 M
	Channel		180.8 G	33.3 M
	Channel + MMTM		180.8 G	33.4 M
	Channel + Spatial		180.8 G	33.3 M
	Channel + MMTM + Spatial		180.8 G	33.8 M

**Table 5:** Object detection performance according to the division of dominance. *C* is the camera dominance module, *L* is the LiDAR dominance module, *B* is the ‘both’ dominance module. Rule means distinguish the influence of each sensor. D: Daytime, N: Nighttime, F: Fog, NF: Not fog, C: Clear, S: Snow.

Backbone Network	Number of Module	Rule	Camera	LiDAR		Both			Total mAP@[.5]
			D, F	N, C	N, S	D, C	D, S	N, F	
EfficientNet-B3	None	None	0.469	0.402	0.436	0.409	0.444	0.407	0.414
	Two Module (*C*, *L*)	D, N	0.382	0.383	0.401	0.371	0.372	0.396	0.371
	Two Module (*C*, *L*)	F, NF	0.391	0.344	0.368	0.367	0.378	0.372	0.362
	Three Module (*C*, *L*, *B*)	D, N, F, NF	0.448	0.417	0.436	0.422	0.448	0.434	0.427

**Table 6:** Object detection performance according to module configuration. Network backbone use EfficientNet-B3. D: Daytime, N: Nighttime, C: Clear, F: Fog, S: Snow.

Number of Module	Module Configuration	Camera	LiDAR		Both			Total mAP@[.5]
		D, F	N, C	N, S	D, C	D, S	N, F	
Three Module (*Camera*, *LiDAR*, *Both*)	Channel	0.355	0.389	0.303	0.325	0.361	0.393	0.365
	Channel + MMTM	0.274	0.377	0.405	0.276	0.295	0.371	0.333
	Channel + Spatial	0.385	0.374	0.401	0.331	0.358	0.381	0.362
	Channel + MMTM + Spatial (Propose)	0.448	0.417	0.436	0.422	0.448	0.434	0.427

## Data Availability

Not applicable.
